# Non-invasive ultrasound assessment of chronic liver disease: current position and future directions for a “one-stop” liver ultrasound approach

**DOI:** 10.1186/s13244-026-02279-4

**Published:** 2026-04-27

**Authors:** Paul S. Sidhu, Mustafa Secil, Dirke-Andre Clevert, Adrian K. P. Lim, Maciej Piskunowicz, Paolo Ricci, Thomas Fischer, Vladimir Mitkov, Vito Cantisani, Caroline Ewersten, European Society of Radiology (ESR)

**Affiliations:** 1 Department of Imaging Sciences, School of Biomedical Engineering and Imaging Sciences, Faculty of Life Sciences and Medicine, King’s College London, London, UK; 2 Department of Radiology, King’s College Hospital NHS Foundation Trust, London, UK; 3 Department of Radiology, Faculty of Medicine, Dokuz Eylul University, İzmir, Türkiye; 4 Interdisciplinary Ultrasound-Center, Department of Radiology, University of Munich, Grosshadern Campus, Munich, Germany; 5 Department of Imaging, Imperial College Healthcare NHS Trust & Department of Digestive Diseases, Reproduction and Metabolism, Imperial College London, London, UK; 6 Radiology Department, Medical University of Gdansk, Gdansk, Poland; 7 Unit of Emergency Radiology, AOU Policlinico Umberto 1 & Department of Radiological, Oncological and Pathological Sciences, Sapienza University of Rome, Rome, Italy; 8 Department of Radiology, Interdisciplinary Ultrasound Center, Campus Charité Mitte, Charité, Universitätsmedizin Berlin, corporate member of Freie Universität Berlin, Humboldt-Universität zu Berlin, Berlin Institute of Health, Berlin, Germany; 9 Diagnostic Ultrasound Department, Russian Medical Academy of Continuous Professional Education, Moscow, Russia; 10 Bessa Department University Sapienza of Rome, Rome, Italy; 11 Department of Radiology, Copenhagen University Hospital, Rigshospitalet, Copenhagen, Denmark; 12 European Society of Radiology, Am Gestade 1, Vienna, Austria

**Keywords:** Ultrasonography, Chronic liver disease, Contrast enhanced ultrasound, Elastography, Fat quantification

## Abstract

**Abstract:**

The integration of the multitude of ultrasound techniques into a “one-stop” liver clinic model will revolutionize the management of liver diseases. This approach streamlines patient care by providing immediate imaging assessment, facilitating prompt diagnosis, and expediting treatment plans. The traditional ultrasound methods of B-mode imaging and Doppler techniques have been supplemented by the newer techniques of tissue elastography, fat quantification, and contrast-enhanced ultrasound—termed multiparametric ultrasound. The deployment of these techniques to establish in more detail the underlying status of liver disease has been profound. The encompassing ultrasound techniques have allowed the ultrasound practitioner to establish a comprehensive assessment of liver disease, allowing further accurate management, and negating the need for additional, often more expensive, imaging to establish the diagnosis. This paper explores the implementation, benefits, and challenges of ultrasound-based one-stop liver clinics, emphasizing their impact on patient outcomes and healthcare efficiency. A detailed assessment of the techniques and their position in the diagnostic armamentarium is reviewed with a comprehensive overview established.

**Critical relevance statement:**

Multiparametric liver ultrasound integrating B-mode, Doppler, CEUS, elastography and fat quantification provides a practical, low-cost one-stop pathway for staging chronic liver disease, assessing portal hypertension surrogates and characterizing incidental lesions, thereby speeding up treatment.

**Key Points:**

- Ultrasound is the first-line imaging investigation for liver disease, with established criteria on B-mode imaging for steatosis and cirrhosis.
- Multiparametric ultrasound integrates morphology, hemodynamics, fibrosis, steatosis, and lesion assessment.
- A one-stop liver ultrasound clinic accelerates decisions and reduces additional imaging.

**Graphical Abstract:**

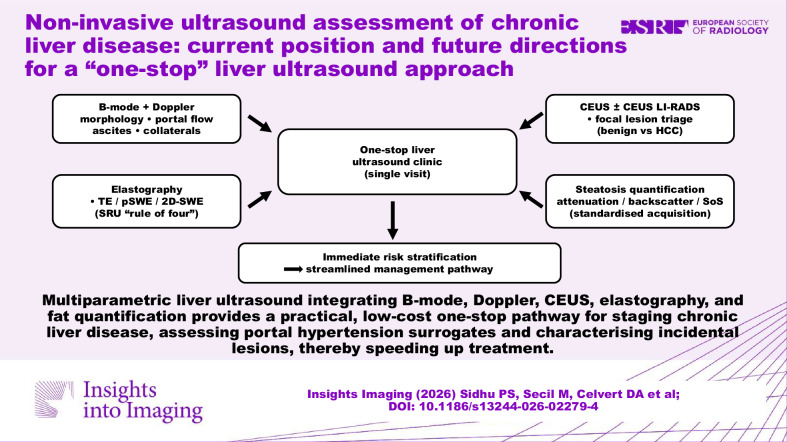

## Introduction

The establishment of ultrasound in clinical practice was the earliest method of liver imaging that enabled, in real time, details that had not previously been apparent with contemporary radiological assessment, including gallstones [1], focal liver lesions, liver abscess [2], and liver texture [3]. Much of what is now taken for granted was revolutionary when it was first encountered, particularly as the introduction of ultrasound preceded any other form of cross-sectional imaging, as neither CT nor magnetic resonance (MR) imaging was available then [4].

Initially, B-mode imaging was an essential tool for many radiology departments in the 1980’s. The subsequent introduction of Doppler techniques, first spectral and subsequently color techniques, provided an immediate leap into better assessment of vascular disease. Recently, this has been further refined with the introduction of extremely sensitive color Doppler techniques able to visualize low-velocity flow, termed microvascular imaging, with the ability to ascertain detailed blood flow within focal liver lesions [5]. The technique of Doppler ultrasound transformed the management of liver transplant assessment; the ability to confirm hepatic artery patency was fundamental to patient management [6, 7].

Following these developments, the introduction of microbubble contrast agents, initially as a “Doppler rescue” agent, allowed for the assessment of the ‘difficult’ portal vein, hepatic artery, or the peripheral vasculature [8–11]. The major development for microbubble contrast agents was the advent of harmonic imaging techniques and the ability to visualize microbubbles with unique temporal and spatial resolution, allowing for the characterization of focal liver lesions [12]. This technique is now mainstream, with guidelines detailing the level of evidence for the efficacy of using microbubbles in the assessment of focal liver lesions [13].

Recent additions to the armamentarium of ultrasound techniques for liver assessment is the ability to accurately assess degrees of liver fibrosis by measuring shear wave velocities, and relating this to established histological criteria [14, 15]. More recently, liver fat quantification, using different techniques, is emerging to challenge the established non-invasive technique of MR imaging [16, 17]. The accuracy of the various ultrasound techniques, using MR imaging as the reference standard, is robust.

All these different aspects of an ultrasound examination, combined together as multiparametric ultrasound [18], have moved liver ultrasound from the basic B-mode and color Doppler ultrasound examination into a comprehensive examination with multiple facets of information, perhaps negating the need for other imaging tests. This article investigates the current understanding of the capabilities of a liver ultrasound examination and takes it further by envisaging a one-stop clinic that serves all the requirements of the clinical referral. In the sections that follow, we discuss conventional B-mode/Doppler and contrast-enhanced ultrasound, ultrasound-based elastography and fat quantification, developing non-invasive approaches to portal hypertension, and an implementation framework for a one-stop liver ultrasound clinic.

## Ultrasound modalities

### B-mode, Doppler, and contrast-enhanced ultrasound

**Fig. 1:**
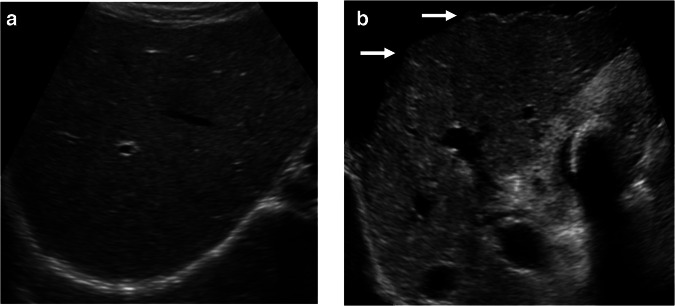
Normal and coarse liver parenchyma. **a** A longitudinal view of the right liver demonstrates the normal smooth homogenous appearances of a normal liver. **b** A longitudinal view in advanced cirrhosis with a coarse heterogeneous appearance to the right liver lobe. Ascites allows identification of the irregular nodular surface of the liver (arrows)

B-mode ultrasound (US) can be used to assess liver parenchymal and surface alterations in chronic liver disease (CLD) [19, 20]. The classical findings include the loss of the homogeneous, smooth echotexture of the liver parenchyma in the early period, followed in time by coarsening of the appearance with variable granular or nodular changes [21–23] (Fig. 1). Subcapsular nodularity causes micro-undulation of the margins and the loss of sharpness of the edges. The loss of smoothness of the liver surface, demonstrated as subcapsular nodularity or tiny undulations, is a reliable finding when identified, achieving a sensitivity and specificity of 0.88 [23]. Parenchymal texture (categorized as fine, mildly coarse, and highly coarse) has been shown to correlate with the degree of fibrosis [21, 23]. Alteration of the hepatic vein delineation is also a reliable finding to show the nodularity within the liver [24].

**Fig. 2:**
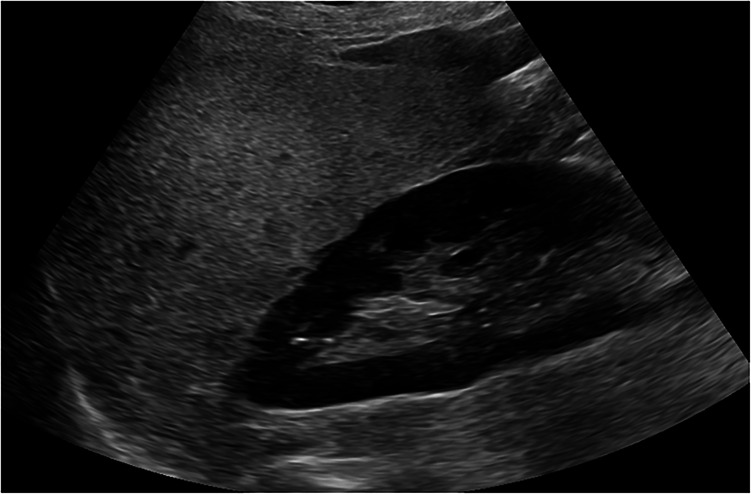
Hepatic steatosis on B-mode. The ‘classical’ B-mode view of the right liver lobe and right kidney for assessing the degree of fatty change of the liver. This is subjectively assessed and would be nominally termed ‘severe’ fatty change. A hepato-renal index was calculated at 6.49, and a quantitatively assessed fat content was estimated at 18.7% in this patient (not shown)

However, in the early stages of disease, these findings are neither straightforward nor simple to recognize on B-mode US. In particular, steatohepatitis with fatty infiltration, may be indistinguishable from just simple fatty infiltration based on B-mode US findings alone (Fig. 2). The atrophy of the central lobes (segment 4 and medial part of segment 8), hypertrophy of the lateral parts of the liver and caudate lobe, and equalization of left and right lobe ratio are well-known late-term findings of CLD. Subjective assessment of the degree of fatty infiltration of the liver, using comparison of the liver to the adjacent reflectivity of the kidney, blurring of vessels, among other features, is often variable, although useful in expert hands [25]. However, when evaluation criteria are standardized, qualitative grayscale ultrasound can be accurate for detecting and grading hepatic steatosis [26].

Radiomics has also been applied to the assessment of the liver parenchyma for fibrosis and cirrhosis with good diagnostic accuracy, but the varied methodologies in numerous studies render radiomics as a yet-to-be-established clinical ultrasound tool [27, 28].

Doppler ultrasound of the liver vessels has been investigated as a potential additional tool for diagnosing CLD [29–31]. However, studies regarding the parameters of the portal veins or hepatic arteries, for example, measured absolute velocities, velocity ratios, etc., have failed to show reliable reproducibility or useful correlation with the diagnosis or the severity of the disease [19, 29]. Hepatic vein waveforms have also been shown to represent inconsistent changes in CLD [31]. The most reliable Doppler findings include the demonstration of portosystemic collaterals, “to-and-fro” or reverse flow in the portal vein, observed together with morphological findings in the liver [30].

The spleen is a secondarily involved organ in the course of CLD, owing to the progressive increase in pressure of the portal system. The enlargement of the spleen and increased diameter of the portal venous system are common findings. Splenoretroperitoneal, splenorenal collaterals, para-esophageal and gastric varices may be observed in late CLD [30, 32]. B-mode ultrasonography can reliably detect clinically relevant ascites, a hallmark of hepatic decompensation, and an important consideration when choosing and interpreting elastography techniques (transient elastography may fail in the presence of ascites).

The studies on contrast-enhanced US (CEUS) in CLD have shown that the transit time of microbubbles is shortened in patients with cirrhosis when compared to non-cirrhotic CLD and normal subjects [33–37]. Hepatic vein arrival time cut-off value of 17 s has been proposed to exclude liver cirrhosis with 91.1% sensitivity and 93.6% specificity [37]. However, CEUS is not widely used for the diagnosis of CLD [19] but is well established in the assessment of any incidental focal liver lesion detected, recognized by the classification system of the American College of Radiology as the Contrast-Enhanced Ultrasound Liver Imaging Reporting and Data System (CEUS-LIRADS) system in CLD [38]. The ability to immediately characterize a focal liver lesion with a CEUS examination is an obvious time-saving procedure, ultimately benefiting patient management [13, 39].

#### Summary

1. B-mode ultrasound provides a reliable examination of liver parenchyma for chronic liver disease and is able to identify texture changes, nodularity, and focal lesions.
2. Liver parenchyma radiomics is reliable for liver fibrosis but is not yet a clinical tool.
3. B-mode ultrasound is able to identify the presence of ascites and measure spleen length reliably.
4. Qualitative B-mode ultrasound detects and grades steatosis when criteria are standardized, and quantitative fat metrics improve reproducibility.
5. Portal vein, hepatic artery and hepatic vein Doppler spectral waveforms and velocities alter in CLD but are not reliable measurements. Identification of varices on color Doppler is useful.
6. Contrast-enhanced ultrasound transit time is not reliable to assess hepatic artery-hepatic vein transit time for the presence of fibrosis or cirrhosis.
7. Contrast-enhanced ultrasound of focal liver lesions is reliable.

### Tissue elastography

It is particularly important to detect the early stages of liver fibrosis that are still reversible. Replacement of liver tissue by connective tissue usually affects the entire organ but may vary in severity and distribution, often patchy. This leads to a very heterogeneous appearance of the disease, which causes well-known sampling errors in liver biopsies, previously the reference standard for clinical management [40, 41]. With the invasive nature of liver biopsy, associated morbidity and mortality, coupled with the need to serially measure the progress of liver fibrosis, alternative non-invasive techniques were developed using imaging techniques.

The concept of relating liver stiffness to the degree of fibrosis or cirrhosis using ultrasound was first accomplished using a vibration technique to generate a shear wave in the liver, measured in kilopascals (kPa) [42], and is the most established and widespread elastography technique for liver fibrosis, transient elastography commonly known by the trade name Fibroscan™ (Echosens) [43, 44]. This device introduces short mechanical shear wave impulses into the liver and measures the shear wave speed (converted to KPa and later to stiffness) in a relatively small tissue volume. The size of this sampling volume, the depth of wave penetration and the presence of ascites have been stated as limitations of transient elastography. Nevertheless, this has led to the widespread application in clinical practice, with the technique used to assess CLD as part of the work-up for assessment, obviating the need for liver biopsy in many cases. The volume of liver assessed by liver elastography is significantly larger than a liver biopsy sample [45].

**Fig. 3:**
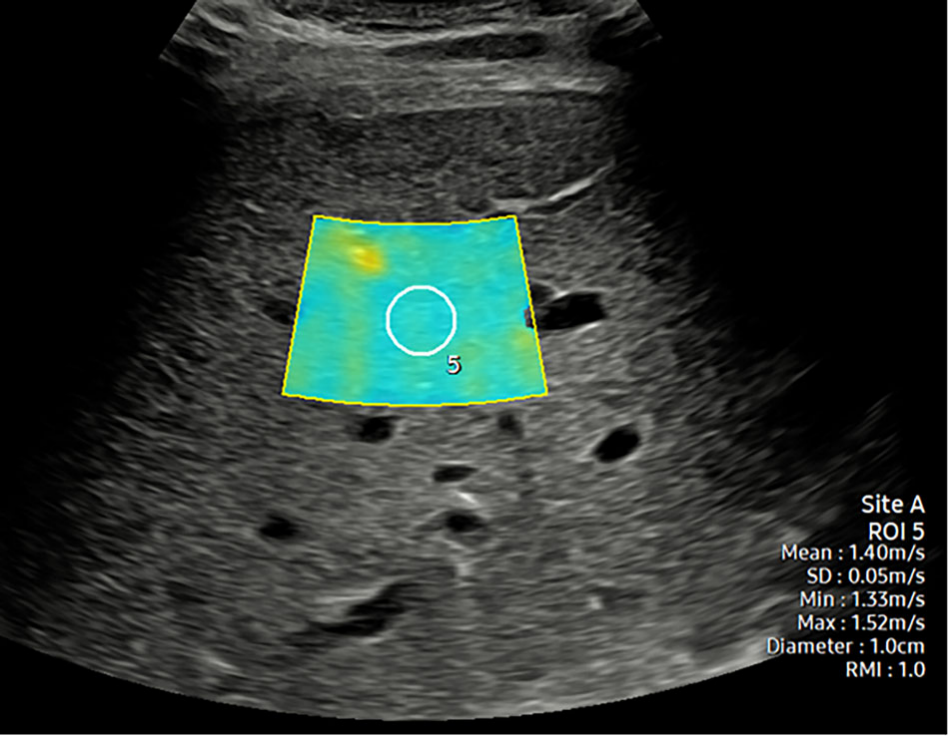
2-D shear wave elastography. A region of interest (ROI) is placed at the optimal position in the right lobe of the liver at a pre-defined level, in suspended respiration. The RMI measure (1.0 in this example) defines the reliability of the measurement, a further ROI is placed and a measure obtained. This can be repeated for the desired number of measurements

Considerable progress has been achieved in the analysis of fibrosis by ultrasound techniques, including the use of shear wave generation by the acoustic radiation force impulse (ARFI) technique, eliciting a shear wave velocity in meters/second, and documented either as a point shear wave elastography (pSWE) or in a two-dimensional shear wave elastography (2D-SWE) method (Fig. 3) [46]. These techniques cover significantly larger volumes and provide a good correlation with morphology even in patients who are difficult to sonicate [15]. Magnetic resonance elastography currently represents the most accurate method with coverage of the entire organ but is restricted by cost and availability [47, 48].

Numerous studies have now established the accuracy of the ultrasound-based shear wave elastography techniques, with evidence at least demonstrating non-inferiority to the transient elastography (Fibroscan™, Echosens) technique and advantageous in patients deemed unsuitable for the transient elastography examinations, most notably in the presence of ascites [49–51]. Both pSWE and 2D-SWE show comparable results when assessing fibrosis [52, 53]. There are differences in the measurement results between machines [54], with authors suggesting the same machine be used in longitudinal assessments in the same patient, with manufacturers working toward rectifying these discrepancies [55–58]. Using the measurement of spleen stiffness as a surrogate marker for the presence of portal hypertension is another use of these techniques when evaluating CLD [59].

For acoustic radiation force impulse (ARFI)-based pSWE and 2D-SWE, the Society of Radiologists in Ultrasound (SRU) consensus update proposes a vendor-neutral “rule of four” (5–9–13–17 kPa to approx. 1.3–1.7–2.1–2.4 m/s) in patients with viral hepatitis and metabolic dysfunction-associated steatotic liver disease (MASLD), to stratify the likelihood of compensated advanced chronic liver disease, identify values suggestive of clinically significant portal hypertension, and define ranges in which confirmatory testing is appropriate [56].

Previously, with pSWE, ten measurements from the right lobe of the liver under controlled acquisition (suspended breathing, fasting) were recommended, but further studies indicate that a minimum of six measurements are equally accurate [60], and with 2D-SWE, this may be less than five measurements. Workflow is an important aspect of any ultrasound examination, and any reduction in the acquisition time is helpful. Further developments by the manufacturers have progressed measurement acquisition; a single acquisition for pSWE elastography can acquire 15 measurements and calculate the appropriate measurement to be reported with the inter-quartile range (Supplementary Fig. 1). With the development of deep learning techniques, the speed of measurement and calculation will further improve.

Currently, liver biopsy is the only reliable method for the evaluation of necroinflammation in the liver. Tissue viscosity can either be quantified based on shear wave attenuation or by measuring the dispersion of stiffness over excitation frequency, termed shear wave dispersion (SWD) [61]. Shear wave velocity from 2D-SWE analyses the shear wave velocity frequency-dependent variation, which is related to liver viscosity. This measures how fast shear wave speed increases with frequency; a steeper slope implies higher viscosity, and this may be related to the degree of liver inflammation [62]. Ultrasound-based viscosity measurements for inflammation could play an important role in the future, distinguishing MASLD from metabolic dysfunction-associated steatohepatitis (MASH) [63]. First approaches, implemented in high-end sonography devices, exploited, in addition to shear wave speed, shear wave attenuation to quantify hepatic steatosis and shear wave dispersion slope to detect a possible inflammatory component [64]. There are still conflicting results, because some studies have not found a correlation with necroinflammation, and some others showed suboptimal performance [62, 65, 66] (Supplementary Fig. 2).

#### Summary

1. The standalone transient elastography device is accurate for measuring liver stiffness, related to the degree of fibrosis and has altered clinical practice in chronic liver disease.
2. The transient elastography device makes measurements of stiffness in kPa; ultrasound-based methods also give measurements in m/s, the parameter derived from liver stiffness.
3. Ultrasound-based techniques generating shear waves in the liver are a later development and can be measured using two methods: pSWE and 2D-SWE, with the latter measuring a larger volume.
4. The techniques are incorporated into the ultrasound machines and can be used in conjunction with all other ultrasound techniques, allowing accurate placement of the region of interest in the liver or spleen, even in the presence of ascites.
5. There are differences in the measurements between different manufacturers, and this is being addressed.
6. Technical improvements will allow measurements to be obtained rapidly and accurately, with an improvement in workflow.
7. Shear wave dispersion calculations may aid the diagnosis of underlying necroinflammation and identify MASH.

### Diffuse liver fat infiltration

The most common diffuse liver disease in Western countries is MASLD [16, 67]. From a clinical point of view, it is important to distinguish MASLD from MASH, as MASH can develop into liver cirrhosis and cancer, with viscosity measurements potentially aiding in the differentiation. The number of people affected by MASLD is estimated at up to 30% of the world’s population, with the degree of fatty infiltration unrelated to the progression to MASH, emphasizing the global problem that this represents [68].

**Fig. 4:**
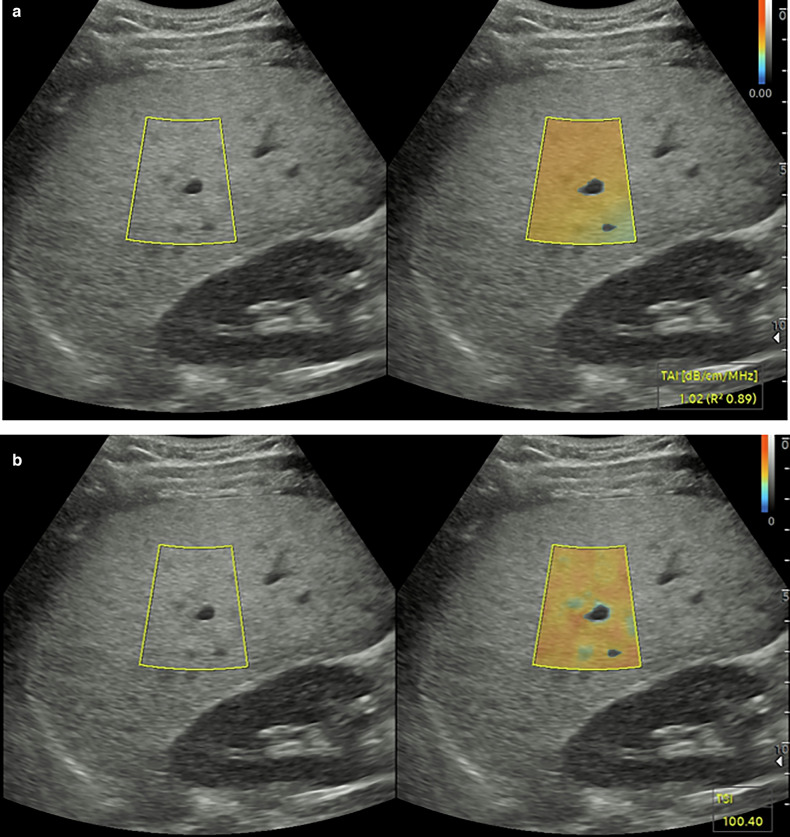
Fat quantification measurements using tissue attenuation and backscatter. The liver is echogenic in comparison to the right kidney, a subjective assessment of fat content. In brief suspended respiration, the region of interest is placed at least 2 cm beneath the liver capsule. **a** A calculated attenuation measure (TAI™) of 1.02 dB/cm/MHz. **b** A calculated backscatter (TSI ™) of 100.40 was obtained in this liver with hepatic steatosis

The ability to quantify the fat content of the liver on ultrasound is a new development that has the potential to become the main technical development that will make a difference to liver imaging [69]. The first method for evaluating fatty change in the liver was an extension of the transient elastography device (Fibroscan™), with the controlled attenuation parameter (CAP) developed [70]. This gives measurements in dB/m, and has shown good results in assessing fatty liver disease with cut-off levels established for the presence of different degrees of fat content of the liver [71]. There are three ultrasound methods for the measurement and quantification of fat: sound attenuation, backscatter, and measurement of the speed of sound [17, 72, 73]. The first two techniques, singly or in combination, are on most commercial machines, with the speed of sound technique for fat quantification less widely available (Fig. 4). Termed quantitative ultrasound, this is an analysis of the raw radiofrequency data returned from the tissue, with sound attenuation measured in dB/m/MHz, depending on the transducer frequency, and sound backscatter is related to the attenuation, speed of sound is measured in m/s.

There is an increasing volume of literature on the usefulness of the quantitative ultrasound methods, and the usual comparison imaging technique is the MR proton-derived fat fraction (MRI-PDFF), with good levels of correlation seen [74]. However, similar issues are emerging with the development of quantitative ultrasound of fat content of the liver, as was apparent with elastography; different measurements between manufacturers are again being identified, and the technique needs to be standardized [75, 76]. The ability to quantify fat content as a numerical percentage will become commonplace and enable both the patient and the physician to understand the progression or regression of liver fat content (Supplementary Fig. 3). Technical improvements with machine and deep learning software contributing to the speed of acquisition of the measurements allow for fat quantification to be processed in a few seconds, greatly improving workflow [77].

#### Summary

1. Ultrasound fat quantification is a rapidly developing measurement technique for assessing the degree of hepatic steatosis, with three different techniques available: attenuation, backscatter and speed of sound.
2. A similar procedure to that used in elastography measurements is used; the patient does not need to fast (but desirable if part of a liver ultrasound examination); attenuation and backscatter are the predominant techniques.
3. Standardization of assessment is needed; manufacturers’ techniques need streamlining to avoid differences in quantification measurements.
4. Presentation of the results in percentage terms, similar to MRI-PDFF, will be useful for clinical management.
5. Workflow improvements are preceding a full investigation into the accuracy of these new fat quantification techniques.

### Non-invasive ultrasound assessment of portal hypertension

The assessment of the presence and severity of portal hypertension in CLD is of paramount importance for patient management, with the measurement of the hepatic venous portal gradient (HVPG) in the hepatic vein considered the reference standard; an invasive procedure that is unsuitable for regular assessment [78–80]. An inexpensive, non-invasive, and accurate method for the assessment of portal hypertension would be desirable [81].

The application of ultrasound Doppler techniques in CLD, examining the portal vein, hepatic veins, and hepatic artery, has been used in order to identify CLD [29–31]. Color and spectral Doppler assessment of the portal vein, and size of the portal vein have been used as surrogate markers for the presence of possible portal hypertension, with the addition of CEUS aiding the differentiation of thrombus (both bland and malignant) from low portal vein flow [10, 82, 83]. These surrogate techniques are easily incorporated into the ultrasound examination but are unable to quantify the HVPG.

A further surrogate marker for the presence of portal hypertension is the application of elastography to assess the splenic stiffness and relate this to the possible presence of significant portal hypertension and the presence of varices [46, 59, 84]. This has been successfully used to predict the presence of portal hypertension in a number of studies as well as to predict the presence of significant varices [85–87]. A combination of liver and spleen elastography, as well as spleen size, may also be a useful tool for the presence of significant portal hypertension [88]. This aids in the evaluation of the presence of portal hypertension but remains a surrogate marker.

A new technique using CEUS evaluates the subharmonic pressure differences between the hepatic and portal veins to estimate the gradient and assess portal hypertension, termed subharmonic aided pressure estimation (SHAPE) [89]. The principle of this technique is that the received subharmonic frequency amplitude of the ultrasound contrast agent relates linearly to the hydrostatic pressure measured in the hepatic and portal veins (provided the acoustic pressure is sufficient for the bubbles to act as non-linear oscillators). This allows for an estimation of the HVPG, similar to measurements conducted with hepatic wedge pressure techniques in interventional radiology (Supplementary Fig. 4) [90, 91]. This technique currently works best with Sonazoid™(GE HealthCare), but studies with Definity™ (Lantheus Medical Imaging) and SonoVue™ (Bracco SpA) have also been reported [92]. SHAPE is likely to become a useful clinical tool and may replace invasive methods of measuring the HVPG, allowing for timely serial measurements for portal hypertension in CLD, both in adults and children [93].

#### Summary

1. Portal vein Doppler ultrasound techniques can assess portal vein blood flow and suggest the presence of hypertension, with spleen size confirmatory.
2. Color Doppler and CEUS can assess flow direction in the portal vein, with CEUS helping to identify bland or malignant portal vein thrombosis.
3. Elastography techniques in the spleen have been used as a surrogate predictive measure for the presence of portal hypertension and varices. This has been used in combination with liver elastography and spleen size to improve the determination of the presence of portal hypertension.
4. Non-invasive contrast techniques measuring pressure in the hepatic and portal veins, termed SHAPE, will be able to measure HVPG accurately.

### Assessment of the incidental focal liver lesion

During the ultrasound examination for CLD, often as part of screening for the development of a hepatocellular carcinoma (HCC), a ‘new’ incidental focal liver lesion is discovered. The traditional pathway is for further assessment with either CT or MR imaging to further characterize a potential HCC. Most surveillance programs for the development of HCC in hepatitis follow-up rely on ultrasound as a readily available, cost-effective modality in this context, with the known shortcomings of not visualizing all of the liver and ‘missing’ ultrasound invisible lesions [94]. Notwithstanding these shortcomings, if a lesion is identified at the screening ultrasound examination, the examining observer should, as the first-line investigation, use CEUS as the definitive investigation [13].

**Fig. 5:**
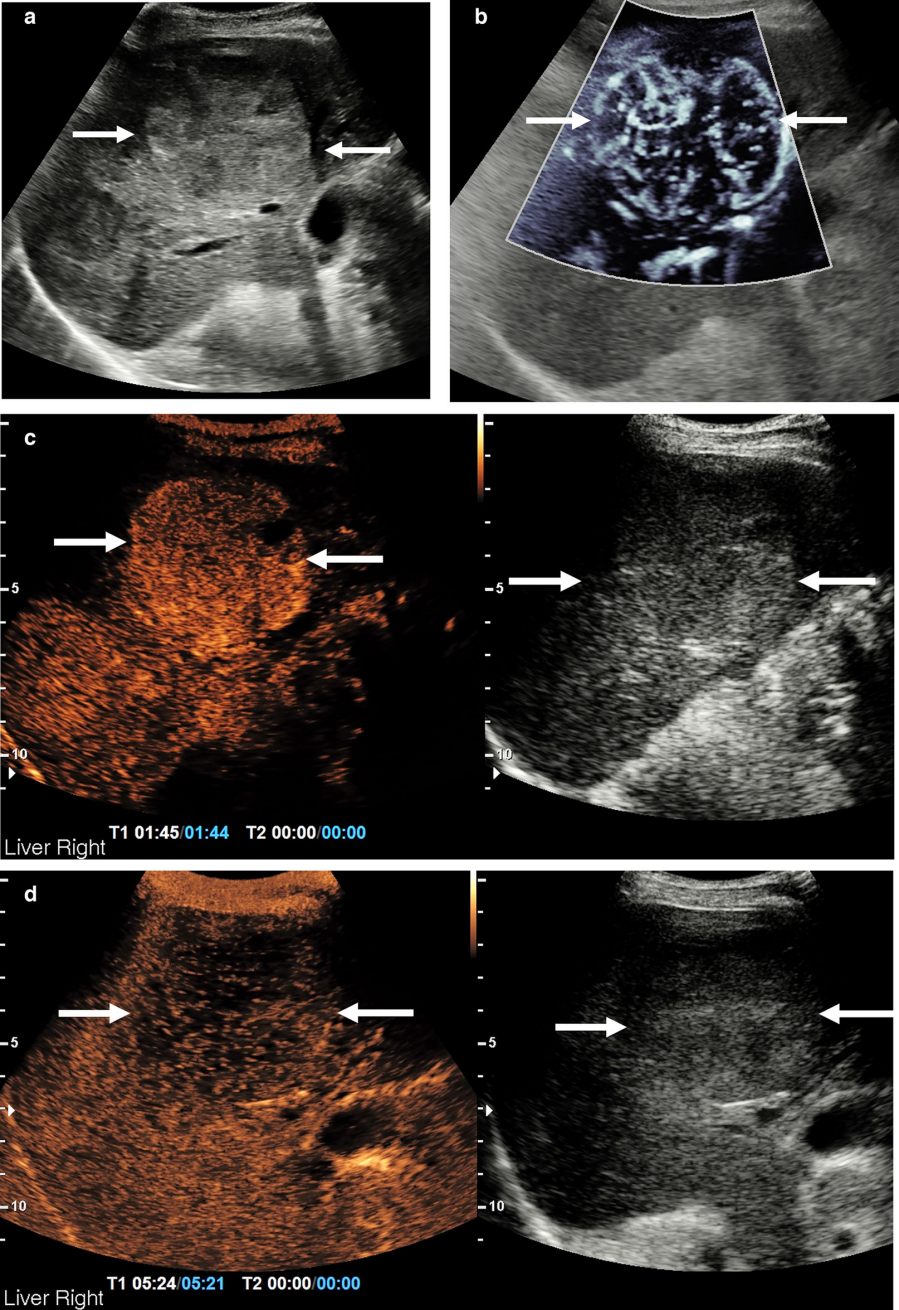
Surveillance ultrasound in a patient with known hepatitis B viraemia lost to follow-up; hepatocellular carcinoma. **a** Multiple focal liver lesions seen in the right lobe of the liver, the largest measuring 58 × 55 mm (arrows), of mixed reflectivity, suggestive of hepatocellular carcinoma. **b** Microvascular imaging demonstrating markedly disordered internal vascularity of the focal liver lesion (arrows). **c** The CEUS examination depicts rapid early arterial enhancement and sustained enhancement at 1 min 44 s. **d** The delayed portal venous phase depicts washout at 5 min 21s, indicating a CEUS-LIRADS 5 lesion

**Fig. 6:**
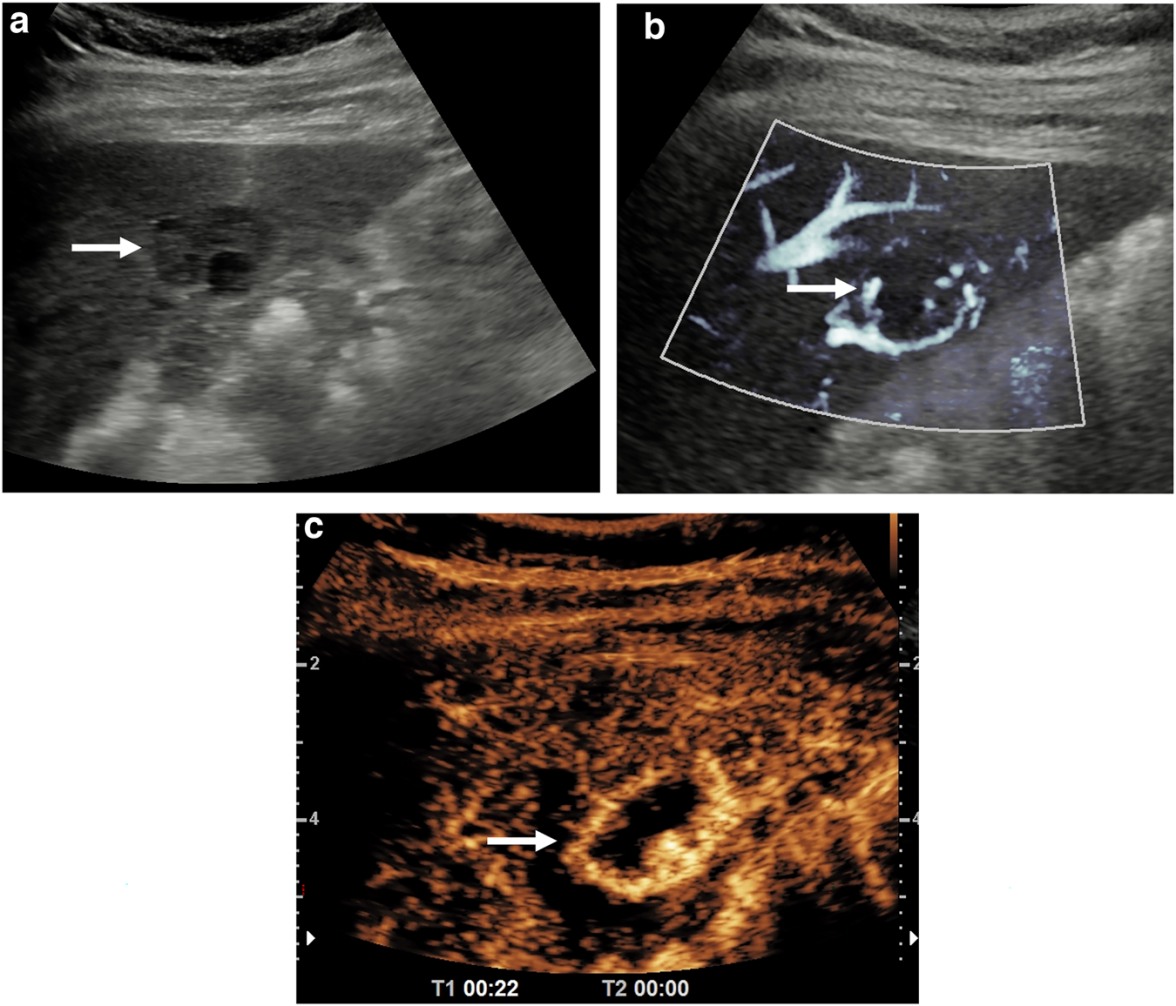
An atypical focal liver lesion on a background of hepatic steatosis, in a patient referred from a primary care physician with abdominal pain; cavernous haemangioma. **a** On the B-mode ultrasound image, a mixed reflective focal lesion (arrow) is noted, without definable characteristic features. **b** Microvascular imaging depicts some “puddling” of vascular flow (arrow), raising the possibility of an atypical haemangioma. **c** The contrast-enhanced ultrasound examination at 22 s, depicts the characteristic globular peripheral enhancement of a haemangioma

The advent of the classification of these lesions using the CEUS-LIRADS protocol allows the immediate triaging of the potential HCC with proven accuracy [95, 96] (Fig. 5). Importantly, CEUS is able to identify and classify the indeterminate lesion with greater certainty than CT or MR imaging [97–101]. Additionally, CEUS identifies benign lesions such as cavernous hemangioma or focal nodular hyperplasia with great certainty, obviating the need for any further CT or MR imaging, both on a background of CLD and in the ‘normal’ liver [102, 103]. In the patient referred from a primary care physician, the ultrasound examination for ‘abnormal liver function tests’ invariably may be normal, or there is hepatic steatosis as a cause. The discovery of an incidental lesion triggers further investigation, which may be a follow-up ultrasound or referral to a specialist clinic for further management, often with further imaging requested. Invariably, these incidental lesions from primary care are benign (up to 90%), and do not require further imaging if confirmed benign [39, 104–108]. The high prevalence of hepatic steatosis makes the classic B-mode appearance of the haemangioma unreliable; however, CEUS can evaluate this rapidly and accurately, preventing unnecessary further investigations (Fig. 6).

#### Summary

1. During an ultrasound surveillance program for assessing chronic liver disease for the development of new focal liver lesions, CEUS should be the next imaging modality and can be performed immediately.
2. CEUS-LIRADS is a comprehensive and accurate method for evaluating the incidental focal liver lesion on the background of chronic liver disease.
3. An incidental focal liver lesion referred from primary care is more likely benign and would benefit from immediate CEUS characterization.
4. With 30% of patients likely to have hepatic steatosis, traditional B-mode ultrasound descriptions of benign focal lesions are inconsistent, requiring CEUS for diagnosis.

## One-stop liver ultrasound clinic

Chronic liver disease is a major healthcare issue, increasing the requirements for accurate and informative imaging. Biochemical testing of liver function introduces patients into the imaging pathway, establishing underlying liver disease and instigating clinical disease management. Ultrasound remains the initial imaging modality. The ability to incorporate as much diagnostic information into one imaging technique, at low cost, is paramount. A comprehensive ultrasound examination is potentially able to offer nearly all the information required to manage liver disease aside from biochemical disease markers. The concept of the one-stop liver ultrasound clinic is nearly a reality, with quantification of portal venous pressures and assessment of necroinflammation the final two pieces of the ‘jigsaw’ yet to fall into place. The ability to assess fibrosis and fat is now well established, on top of the CEUS examinations and the traditional methods of Doppler ultrasound with B-mode evaluation long established. Improvements in imaging and workflow, using ‘artificial intelligence’ (AI) algorithms, will streamline the comprehensive US examination, making it time-efficient. The cost savings of this approach will be the driving force for the implementation of the “one-stop shop” across healthcare systems.

## Supplementary information

ELECTRONIC SUPPLEMENTARY MATERIAL

## Data Availability

Not applicable as this is a review article.
