# Supplementary material for: Non-invasive ultrasound assessment of chronic liver disease: current position and future directions for a “one-stop” liver ultrasound approach
Source: Insights Imaging. 2026 Apr 27;17:120. doi: 10.1186/s13244-026-02279-4 (PMC13121678; doi:10.1186/s13244-026-02279-4)

Non-invasive Ultrasound Assessment of Chronic Liver Disease: Current Position and Future Directions for a "One-Stop" Liver Ultrasound Approach

ELECTRONIC SUPPLEMENTARY MATERIAL

Supplementary Fig. 1
Multifocal Point Shearwave Measurements
The newer techniques of measurement of point shear wave velocities in the liver by the ARFI technique, allows for the simultaneous placement of 15 regions of interest to record liver stiffness in the right liver lobe, and display this in either m/sec or kPa. The most reliable measurements are included in the calculations.

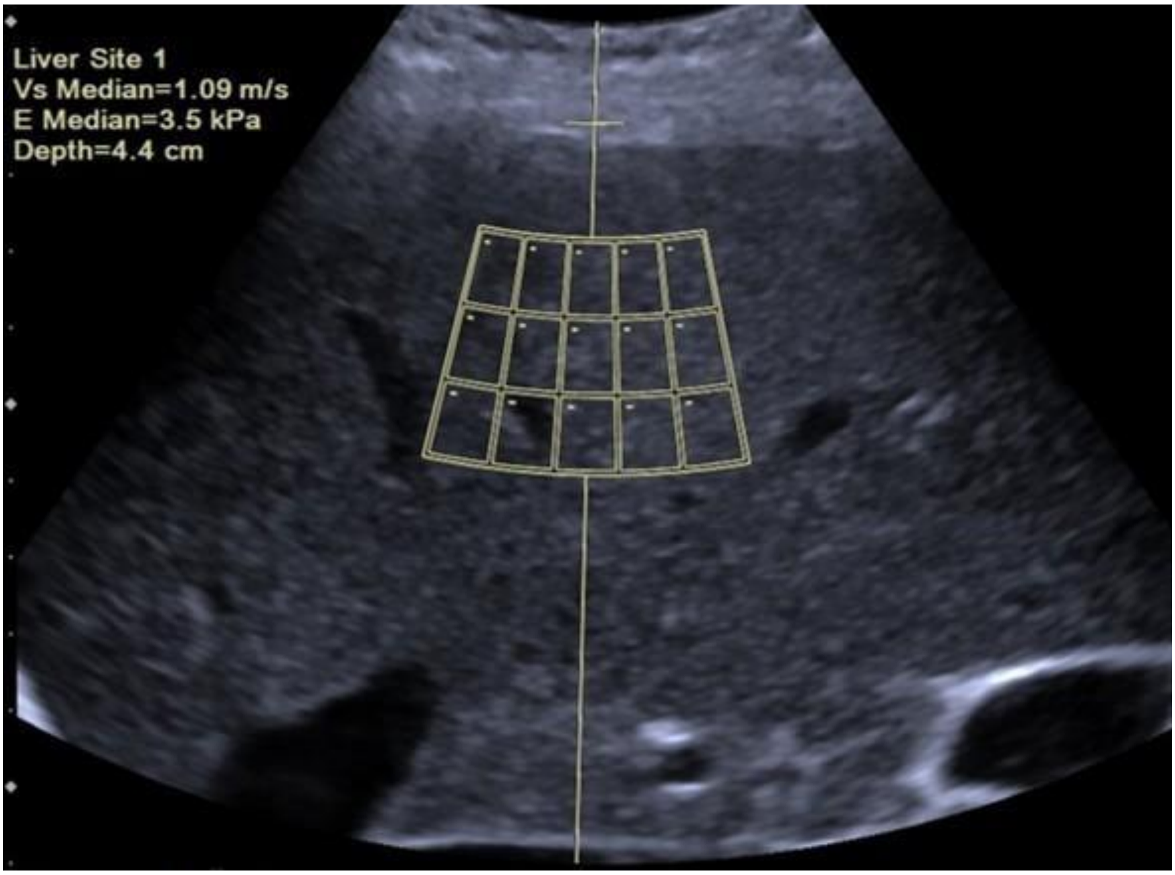

Supplementary Fig. 2
Shearwave Dispersion Slope measurement of Liver Viscosity
The collage of images demonstrates the steps in acquisition of the parameters used to calculate the Shearwave dispersion slope, where faster Shearwave speed increase indicates higher viscosity with steeper slopes possibly correlating with the presence of liver inflammation.

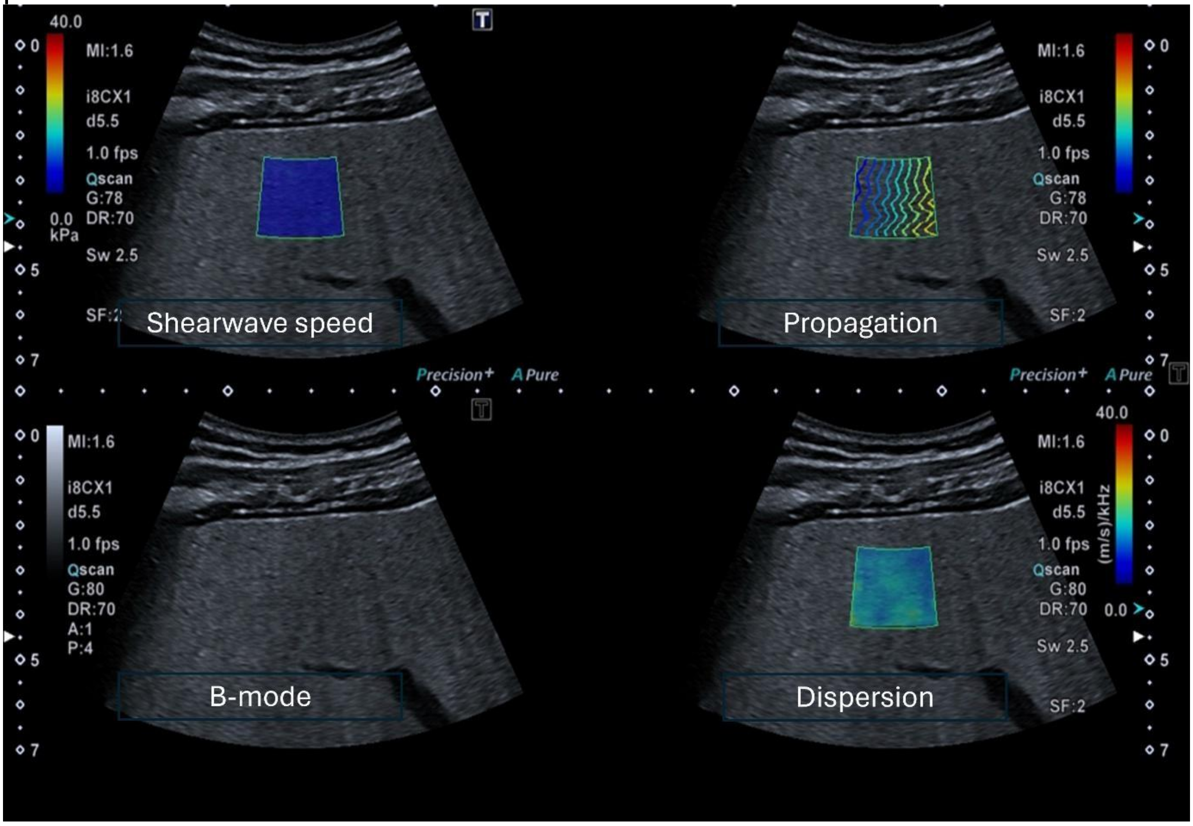

Supplementary Fig. 3
Measurement of Liver Fat as a percentage
A single image of fat quantification using a heat map based artificial intelligence, and deep learning to identify the most suitable area of the liver for quantification of the fat content. The measurements make use of tissue attenuation and backscatter to calculate a fat percentage.

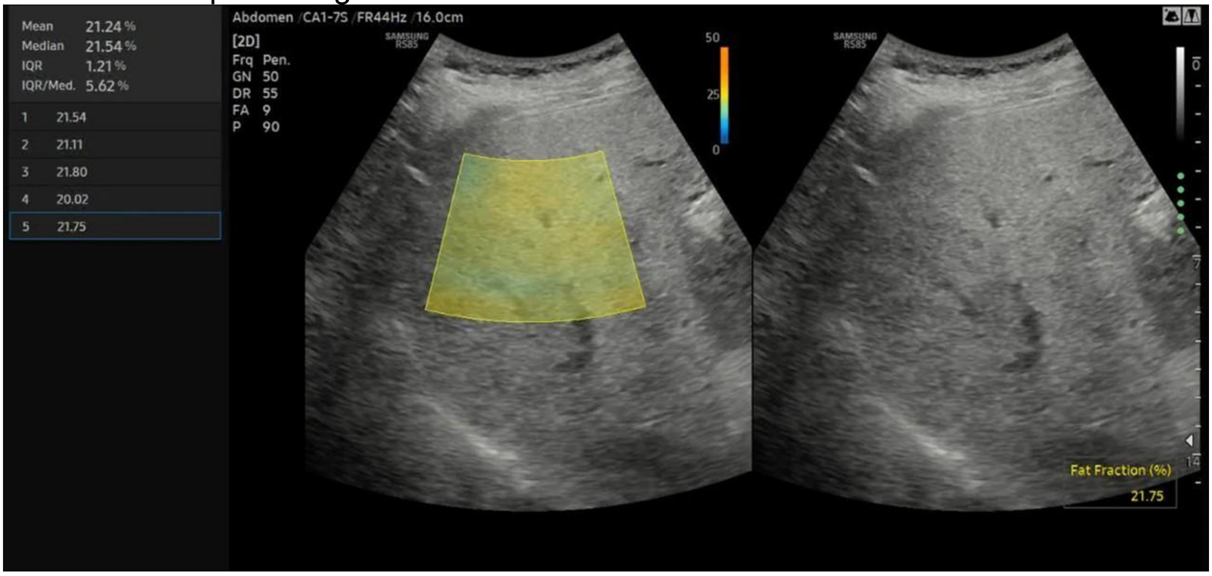

Supplementary Fig. 4
Portal and Hepatic Veins Pressure Measurements
A 50-year-old female with a subharmonic aided pressure estimation (SHAPE) gradient of -3.5 dB and an HVPG of 8 mmHg. The Region of Interest Boxes are placed over the hepatic vein (HV) and the portal vein (PV). (Courtesy of Prof. Flemming Forsberg).

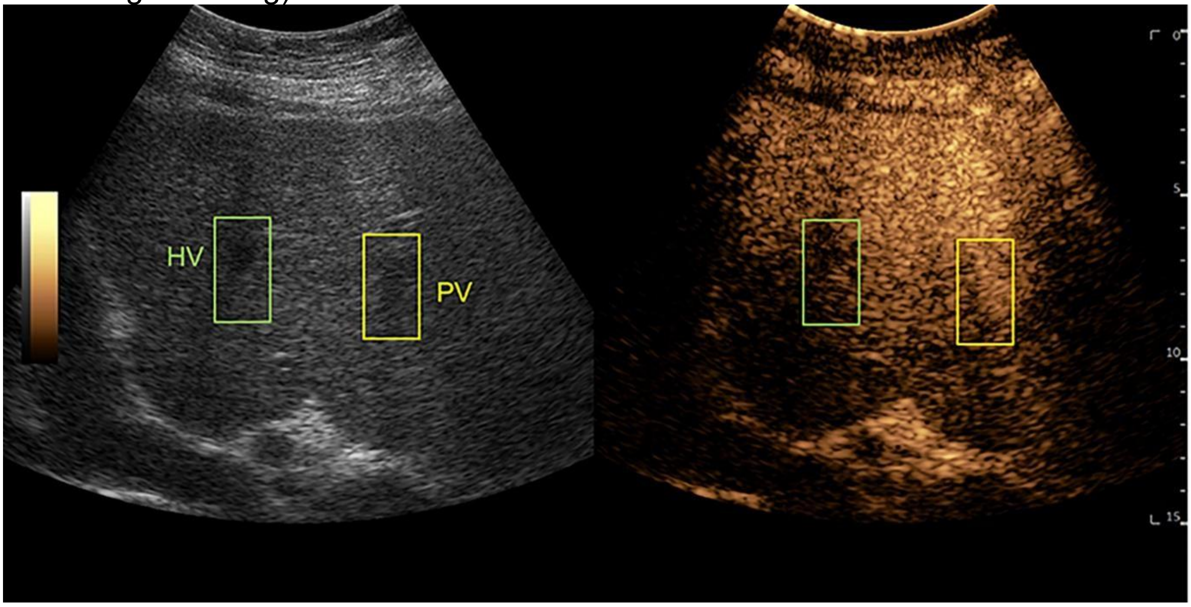

Supplement: Supplementary file 1 — ELECTRONIC SUPPLEMENTARY MATERIAL [file 13244_2026_2279_MOESM1_ESM.pdf]
